# CT–Clinical Dissociation and Focal Obstruction in Feline Chronic Rhinosinusitis: A Pilot Study

**DOI:** 10.3390/vetsci13101120

**Published:** 2026-10-20

**Authors:** Gippeum Lee, Jinhwa Chang, Jeongmin Lee, Kun-Ho Song

**Affiliations:** 1 Department of Veterinary Internal Medicine, College of Veterinary Medicine, Chungnam National University, Daejeon 34134, Republic of Korea; vetmania@cnu.ac.kr (G.L.); memo7677@cnu.ac.kr (J.L.); 2 Korea Animal Medical Center, Cheongju 28651, Republic of Korea; bbuah@hanmail.net

**Keywords:** feline chronic rhinosinusitis, computed tomography, CT–clinical dissociation, focal obstruction, pilot study

## Abstract

Feline chronic rhinosinusitis (CRS) is common and difficult to manage, and the relationship between computed tomography (CT) findings and clinical severity remains unclear. This exploratory retrospective pilot study included 10 cats with chronic upper respiratory signs for at least 4 weeks that underwent CT of the nasal cavity and paranasal sinuses at a single referral hospital. Newly developed, unvalidated semiquantitative scores graded CT severity (0–12) and clinical severity (0–12), and cats were grouped into four provisional clinicoradiologic patterns using integrated CT, clinical, and cytologic findings and, where available, microbiologic findings. CT scores ranged from 2 to 10 (median 8.0) and clinical scores from 4 to 11 (median 6.5), without a consistent monotonic association (Spearman *r*_s_ = 0.250, 95% bootstrap confidence interval (CI) −0.558 to 0.885, *p* = 0.486). The provisional patterns were active/exudative (*n* = 2), structural/remodeling (*n* = 4), obstructive (*n* = 3), and immune/mucosal (*n* = 1). Focal obstruction defined both the obstructive pattern and the conceptual surgical candidacy categories, so their overlap is by design; of interest is that cats with the highest CT scores did not necessarily have focal obstruction. No cat underwent surgery. These hypothesis-generating findings require validation in larger prospective studies with validated scores.

## Simple Summary

Cats can develop long-lasting inflammation of the nose and sinuses, called chronic rhinosinusitis, which is often difficult to treat and sometimes raises the question of surgery. Veterinarians commonly use computed tomography (CT) to examine the nose and sinuses, but how severe the CT findings appear does not always match how unwell the cat is. In this small pilot study of 10 cats, we explored how CT findings and clinical signs related to one another. The two did not consistently match, and the overall CT score did not identify the cats in which a localized obstruction of sinus drainage was present; this obstruction, rather than the overall CT score, appeared to be the more informative CT feature for considering surgery. We also describe four provisional patterns that combine CT and clinical findings. These observations are preliminary and are intended to guide larger studies rather than to inform treatment decisions directly.

## 1. Introduction

Feline chronic rhinosinusitis (CRS) is a common yet clinically challenging condition characterized by persistent or recurrent nasal discharge, sneezing, and varying degrees of upper airway obstruction [1]. Despite its high prevalence in feline practice, CRS remains a frustrating disease to manage, as many patients show incomplete response to therapy and frequent relapse [1].

Traditionally, feline CRS has been associated with antecedent viral upper respiratory infection, particularly feline herpesvirus type 1 (FHV-1), which is believed to initiate epithelial injury and mucosal dysfunction [1]. However, increasing evidence suggests that persistent viral infection alone does not fully explain the chronicity and variability of the disease [1,2]. Instead, CRS appears to result from a complex interaction between initial mucosal injury, secondary bacterial colonization, impaired mucociliary clearance, and progressive structural remodeling of the nasal cavity and paranasal sinuses [1,3,4].

Recent studies have emphasized the role of structural damage in disease progression. Cats that develop CRS at an early age tend to exhibit more severe turbinate lysis and sinus abnormalities, suggesting that disease phenotype may vary depending on age at onset and disease duration [5]. These findings support the concept that feline CRS is not a single disease entity, but rather a heterogeneous spectrum involving varying contributions of inflammation and structural change [1,2].

Computed tomography (CT) has become an essential diagnostic tool for evaluating feline sinonasal disease, enabling detailed assessment of turbinate architecture, sinus involvement, and osseous remodeling [3,6]. However, in clinical practice, clinicians frequently encounter a discrepancy between CT findings and clinical severity [3,6]. Some cats with marked structural destruction exhibit only mild clinical signs, whereas others with minimal CT abnormalities present with severe clinical disease [3]. This mismatch suggests that CT findings may primarily reflect cumulative structural damage, while clinical signs are more closely associated with current inflammatory activity [1,3,6].

The concept that a single disease label may encompass mechanistically distinct subtypes has been increasingly recognized in human chronic rhinosinusitis, where the traditional dichotomy of CRS with versus without nasal polyps is being supplemented by phenotype- and endotype-based classification systems [7,8]. Proposed classification frameworks in human CRS increasingly incorporate anatomical distribution of disease (local vs. diffuse) and inflammatory endotype dominance (type 2 vs. non-type 2), with the latter supported by molecular biomarkers such as tissue eosinophil counts, cytokine profiles, and immunoglobulin E [7,8,9]. These endotype-driven approaches have been linked to differential treatment responses and are beginning to inform the use of targeted biologic therapies [9,10]. Notably, the poor correlation between imaging severity and symptom severity has also been well documented in human CRS, reinforcing that structural disease burden and inflammatory activity represent distinct pathophysiologic domains; however, this dissociation has not been adopted as a primary classification axis in human CRS, where endotype classification based on measurable biomarkers is feasible [11]. Whether patterns of CT–clinical dissociation could help characterize feline CRS has not been systematically explored. Because the molecular biomarker infrastructure that enables endotype-driven classification in human CRS is not yet established in veterinary practice, an exploratory approach based on clinically accessible tools—computed tomography and clinical severity scoring—is a reasonable first step to examine the distinction between structural damage and inflammatory activity [7,8,9].

Considering CT–clinical dissociation patterns in feline CRS may have implications for treatment selection. Currently, management of feline CRS is typically multimodal, including antimicrobial therapy, anti-inflammatory or immunomodulatory treatment, and supportive care [1,12,13]. Despite these approaches, treatment response is often incomplete, and a subset of patients becomes refractory [1,12]. Importantly, these therapeutic strategies address different components of a complex and multifactorial disease process, and no single modality is consistently curative [1,2,4,12]. Surgical intervention has been described in selected cases, particularly those involving sinus obstruction or retained intraluminal material, but outcomes are variable and indications remain poorly defined [14,15]. This variability likely reflects differences in underlying disease mechanisms, and highlights the need to better identify patients in whom mechanical obstruction and impaired sinus drainage play a primary role.

Distinguishing structural damage from inflammatory activity may have treatment implications: cats whose disease is primarily inflammatory without focal obstruction may be managed medically, whereas focal obstruction and impaired sinus drainage may warrant consideration of surgery to restore ventilation and drainage, regardless of concurrent inflammatory activity. If surgical consideration depended on CT severity alone, cats with the highest CT scores would uniformly be candidates; clinical experience suggests otherwise, implying that a functional rather than purely quantitative interpretation of imaging may be needed. A major gap in current decision-making is the lack of an approach that links imaging findings with clinical presentation and helps identify cats for which surgery might be considered versus continued medical management [1,3].

Therefore, the objectives of this exploratory pilot study were:

(1) to explore the pattern of relationship between CT-based structural severity and clinical severity in cats with CRS;

(2) to describe provisional clinicoradiologic patterns based on integrated CT and clinical assessment;

(3) to explore whether CT evidence of focal obstruction, rather than overall CT severity, may provide additional information relevant to surgical consideration.

We hypothesized that the discrepancy between CT findings and clinical severity reflects, in part, the distinction between structural damage and inflammatory activity, and that focal obstruction or impaired sinus drainage, rather than overall CT severity, may be the more relevant CT feature for surgical consideration [1,3]. Given the small sample size, retrospective design, and newly developed scores, this study is intended to generate hypotheses rather than to establish a classification or a surgical decision-making framework.

## 2. Materials and Methods

### 2.1. Study Design and Case Selection

This retrospective observational case series was conducted at a single veterinary referral hospital. Medical records were searched for client-owned cats evaluated between February 2024 and February 2026 for chronic upper respiratory signs (including nasal discharge, sneezing, stertor, and nasal obstruction) that underwent CT examination of the nasal cavity and paranasal sinuses. Of 22 cats identified with chronic upper respiratory signs and available CT imaging during the study period, cats were excluded if CT and associated work-up established a diagnosis of neoplasia or laryngotracheal stenosis rather than CRS, or if imaging was of insufficient quality for semiquantitative scoring. The remaining 10 cats with a final diagnosis of CRS met the following inclusion criteria for this case series: (1) persistent or recurrent upper respiratory signs for a minimum of 4 weeks’ duration; (2) CT examination of the nasal cavity and paranasal sinuses performed at the referral institution; and (3) clinical severity assessment documented at or within 2 weeks of the CT examination.

Ten of the 22 screened cats met the inclusion criteria and were included in the final analysis. Breed, age, sex, body weight, clinical history, and results of diagnostic testing (polymerase chain reaction [PCR], cytology, bacterial/fungal culture, histopathology) were extracted from medical records; virological (PCR) results were recorded separately from cytologic findings. Follow-up data, including treatment response and subsequent imaging where available, were also recorded. In all 10 cases, cytologic specimens were obtained via nasal lavage performed as a diagnostic procedure only; nasal lavage was not intended or used as a therapeutic intervention, and cytologic results were available for all 10 cats. Microbiologic testing (bacterial/fungal culture and PCR) was available only in a subset of cats. Histopathology required an additional invasive procedure and was therefore available in only a subset of cases; where available, it was used as supportive evidence for pattern assignment rather than as an independent discriminating criterion.

### 2.2. CT Acquisition and Scoring

CT examinations were performed under general anesthesia with cats positioned in sternal recumbency, using a 16-row multidetector CT scanner (Somatom Emotion, Siemens Medical Systems, Forchheim, Germany) in helical mode before and after intravenous administration of iohexol (Omnipaque 300, GE Healthcare, Shanghai, China; 2.0 mL/kg). Acquisition parameters were 130 kVp, 150 mAs, and pitch 0.9, with 1-mm soft-tissue reconstruction. A novel semiquantitative CT severity scoring system (0–12) was developed for this study, comprising four subscores each ranging from 0 to 3. This system differs from the Lund-Mackay staging system used in human CRS [16], which assigns per-sinus scores of 0–2 based on opacification extent, in three key respects: (1) it incorporates turbinate destruction as a distinct subscore, reflecting the central role of conchal pathology in feline sinonasal disease; (2) it grades intraluminal material by attenuation characteristics rather than by presence or absence of opacification alone; and (3) it includes bony remodeling as a separate domain to capture the osteoproliferative changes characteristic of chronic feline CRS. Content validity was established through iterative review and consensus among the investigators—including a board-certified veterinary radiologist and a small animal internal medicine specialist—with reference to previously published feline CT scoring approaches [3,6]. The four subscores are defined as follows:

• Turbinate destruction (0–3): 0 = normal architecture; 1 = mild, patchy loss; 2 = moderate, partial destruction; 3 = severe, extensive or complete destruction.
• Sinus involvement (0–3): 0 = no sinus abnormality; 1 = unilateral or minimal fluid/fluid-level in one sinus; 2 = bilateral or moderate fluid accumulation; 3 = bilateral, diffuse, or complete sinus opacification.
• Intraluminal material (0–3): 0 = none; 1 = low-attenuation fluid only; 2 = moderate soft tissue density material; 3 = high-attenuation, mineralized, or inspissated material.
• Bony remodeling (0–3): 0 = none; 1 = mild periosteal or endosteal reaction; 2 = moderate osseous proliferation or bone thinning; 3 = severe remodeling, hyperostosis, or osteolysis beyond turbinates.

The presence or absence of focal obstruction was assessed qualitatively as a binary variable based on CT evidence of retained high-attenuation inspissated material within a sinus, anatomical obstruction of the nasopharynx, or other features indicating impaired sinus drainage amenable to surgical intervention.

CT scoring and obstruction assessment were performed by Jinhwa Chang, DVM, PhD (veterinary radiologist), with knowledge of clinical presentation, as integrated assessment was part of the study design; scoring was therefore not blinded; however, obstruction was assessed as a binary variable independently of surgical candidacy classification. In cases with serial CT examinations, the index scan (most recent or most complete evaluation) was used for the primary analysis; follow-up scans were scored using the identical semiquantitative system and compared to the index scan, and CT acquisition parameters were reviewed for consistency across time points. Longitudinal changes were described narratively.

### 2.3. Clinical Severity Scoring

A novel semiquantitative clinical severity scoring system (0–12) was developed for this study, based on the documented clinical assessment at or near the time of CT. Content validity was established through consensus among the investigators (Gippeum Lee, Jinhwa Chang, and Jeongmin Lee), with subscore domains selected to capture the principal clinical manifestations of feline CRS, and the system underwent face validation by two clinicians—Jeongmin Lee, DVM, PhD (small animal internal medicine specialist), and Jinhwa Chang, DVM, PhD (veterinary radiologist)—prior to retrospective application. The scoring system has not been formally validated, and inter- and intraobserver agreement were not assessed. The four subscores are defined as follows:

• Nasal discharge severity (0–3): 0 = none; 1 = intermittent serous or mild discharge; 2 = persistent mucoid or mucopurulent discharge; 3 = profuse purulent discharge, crusting, blood-tinged discharge, or spraying pattern.
• Sneezing frequency (0–3): 0 = none; 1 = occasional; 2 = daily, recurrent; 3 = frequent or severe sneezing, including sleep-disturbing sneezing or repeated sneezing bursts.
• Airflow obstruction/stertor (0–3): 0 = none; 1 = mild, intermittent respiratory noise; 2 = persistent stertor or clinically documented evidence of unilateral nasal obstruction; 3 = marked nasal blockage, sleep disturbance attributable to obstruction, or overt respiratory effort. This subscore reflects clinically documented obstruction (e.g., audible stertor, observed respiratory effort) rather than CT-defined structural obstruction, which was scored separately (Section 2.2).
• Functional/quality-of-life impact (0–3): 0 = no discernible functional impact; 1 = mild impact; 2 = moderate impact requiring ongoing management; 3 = clear impact on daily activity, sleep, appetite, or other important aspects of quality of life, based on contemporaneous medical-record evidence such as sleep disturbance, impaired eating or activity, need for repeated visits or ongoing management, rapid clinical worsening upon treatment discontinuation, or explicit owner-reported deterioration.

Clinical scores were assigned retrospectively from medical records by Gippeum Lee, DVM, MS (feline medicine specialist); the evaluator was not blinded to CT findings, as clinical and imaging data were interpreted together in clinical practice.

### 2.4. Exploratory Clinicoradiologic Pattern Classification

Cases were grouped into provisional clinicoradiologic patterns (provisional phenotypes) based on integrated interpretation of (1) total CT severity score and subscore profile, (2) total clinical severity score, (3) the direction and magnitude of CT–clinical dissociation (CT score minus clinical score), (4) qualitative assessment of focal obstruction on CT, and (5) nasal lavage cytology (available for all cats) and, where available, microbiologic data (PCR results, bacterial/fungal culture). The grouping was performed by consensus among the investigators after reviewing all available data for each case, without blinding. Because the same data were used to define and describe the patterns, the grouping is descriptive and was not validated.

The principal discriminating axes underlying the four provisional patterns were as follows. CT evidence of focal obstruction or impaired sinus drainage was the clearest criterion distinguishing the obstructive pattern from the other three. Among non-obstructive cases, preservation versus destruction of turbinate architecture was the next discriminant: cases with predominant turbinate destruction were assigned to the structural/remodeling pattern, whereas cases with preserved turbinate architecture were considered for either the active/exudative or immune/mucosal pattern. Between these two turbinate-preserved patterns, the level of CT and clinical severity together with the cytologic pattern (neutrophilic versus lymphoplasmacytic inflammation) served as the further discriminating criterion: cases with prominent intraluminal exudate, neutrophilic cytology, and comparatively high clinical severity were assigned to the active/exudative pattern, whereas cases with mild CT and clinical findings and a lymphoplasmacytic cytologic pattern were assigned to the immune/mucosal pattern.

### 2.5. Conceptual Surgical Candidacy Categories

Surgical candidacy categories were defined a priori and conceptually, based on CT evidence of focal obstruction or impaired sinus drainage rather than on the total CT severity score. Cases were categorized as: Medical (no surgical target identified; medical management indicated), Conservative (structural damage present but no focal obstruction; continued conservative management appropriate), Consider surgical intervention (borderline obstructive features; surgical option to be reconsidered if medical management fails), or Surgical candidate (clear obstructive target amenable to surgical drainage or decompression). This logic was grounded in the principle, articulated in clinical practice guidelines for the surgical management of human chronic rhinosinusitis, that sinus drainage obstruction refractory to medical therapy is a principal justification for surgical intervention [17]. Focal obstruction was therefore a defining criterion of both the obstructive pattern and the upper candidacy categories, and the overlap between them is definitional rather than a finding. The question explored with these data was whether the summed CT severity score, which does not itself define obstruction, tracked with obstruction status. No cat in the present series underwent surgery (e.g., frontal sinus trephination); the categories are a conceptual interpretation of CT findings and have not been validated against surgical outcomes.

### 2.6. Statistical Analysis

Given the small sample size (*n* = 10), all statistical analyses were considered exploratory and hypothesis-generating. Spearman’s rank correlation coefficient (*r*_s_) was calculated to assess the monotonic association between CT and clinical severity scores. A bias-corrected and accelerated (BCa) bootstrap 95% confidence interval for *r*_s_ was estimated using 10,000 resamples; the BCa method was selected over the simpler percentile method because it corrects for both bias and skewness in the bootstrap distribution [18,19]. Nonetheless, at *n* = 10, the confidence interval should be interpreted with caution, as coverage of bootstrap confidence intervals—even with the BCa method—is reduced in very small samples [18,20]. Descriptive statistics are reported as median and range (or interquartile range [IQR] where indicated). Correlation coefficients, confidence limits, and *p* values are reported to three decimal places, and medians and interquartile ranges of scores to one decimal place. A *p* value < 0.05 was considered the threshold for statistical significance, with the caveat that the small sample size substantially limits statistical power and increases the risk of both type I and type II error. No adjustment for multiple comparisons was applied, given the exploratory nature of all analyses. Analyses were performed using Python, SciPy, and NumPy, and the results were confirmed using Python 3.13.16, SciPy 1.18.1, and NumPy 2.5.3. 

## 3. Results

### 3.1. Study Population

Ten client-owned cats diagnosed with CRS were included. Breeds comprised nine Korean Shorthair and one British Shorthair. The sample included four spayed females, four neutered males, and two cats of unrecorded sex. Age at the time of CT ranged from 1 to 8 years (median 3.5 years); body weight ranged from 3.0 to 5.77 kg (median 4.85 kg). All cats had persistent or recurrent upper respiratory signs exceeding 4 weeks and underwent CT at a single referral institution.

### 3.2. CT and Clinical Severity Score Distribution

CT severity scores ranged from 2 to 10 (median 8.0; IQR 5.3–10.0) and clinical severity scores ranged from 4 to 11 (median 6.5; IQR 6.0–9.3). The highest CT scores (10/12) were observed in four cats (Cases 2, 4, 6, and 10), and the lowest (2/12) in Case 3. The highest clinical score (11/12) was recorded in Case 1 and the lowest (4/12) in Case 3. Individual case scores and CT subscores are presented in Table 1.

### 3.3. CT–Clinical Correlation

**Figure 1:**
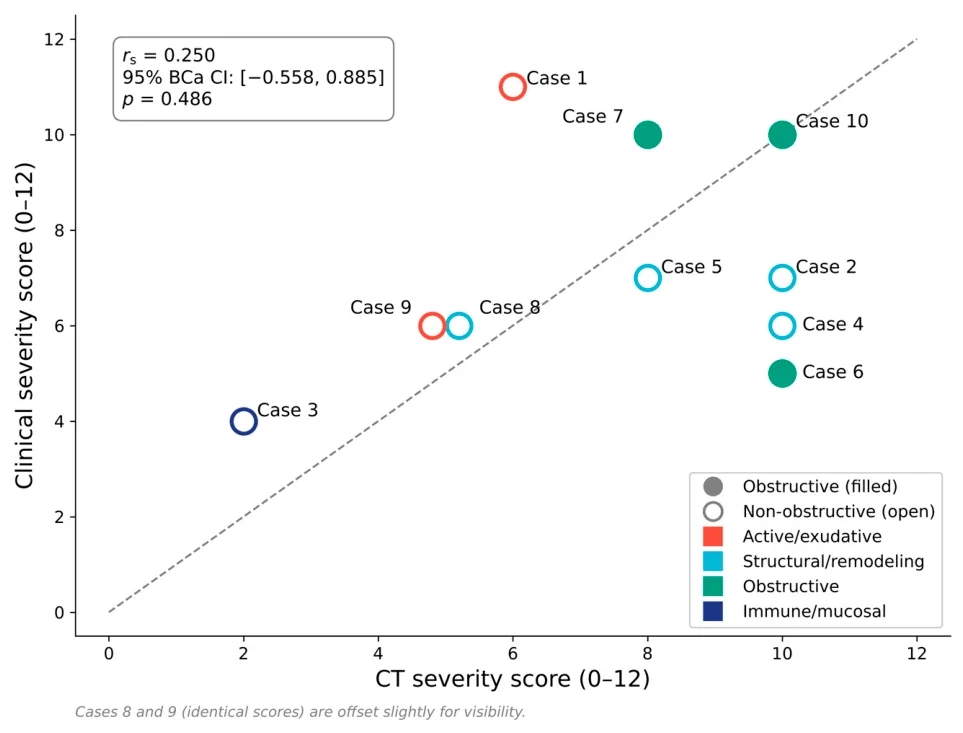
Scatter plot of CT severity scores versus clinical severity scores in 10 cats with chronic rhinosinusitis, colored by provisional pattern and shaped by obstruction status. The dashed line represents the identity line (CT = Clinical). Cases below the line have clinical severity exceeding CT severity; cases above the line have CT severity exceeding clinical severity.

Spearman’s rank correlation between CT and clinical severity was *r*_s_ = 0.250 (*p* = 0.486), with a bootstrap 95% confidence interval of −0.558 to 0.885 (10,000 resamples). This extremely wide interval, spanning nearly the entire possible range of correlation coefficients, precludes interpretation of either the direction or the magnitude of the association and reflects the imprecision of correlation estimates at *n* = 10; the absence of a statistically significant correlation should not be interpreted as evidence of no relationship. Cases were widely dispersed across the CT–clinical spectrum without a consistent monotonic trend (Figure 1).

The direction and magnitude of CT–clinical dissociation varied across cases (CT—Clinical score, Table 1). The difference ranged from −5 to +5. Negative values, indicating clinical severity exceeding CT findings, were most pronounced in Case 1 (−5), the case with the highest clinical score (11/12) but only moderate CT findings (6/12). Positive values, indicating CT findings exceeding clinical severity, were most pronounced in Case 6 (+5) and Case 4 (+4), both with CT scores of 10/12 but relatively mild clinical scores (5/12 and 6/12, respectively).

### 3.4. Provisional Clinicoradiologic Patterns

**Figure 2:**
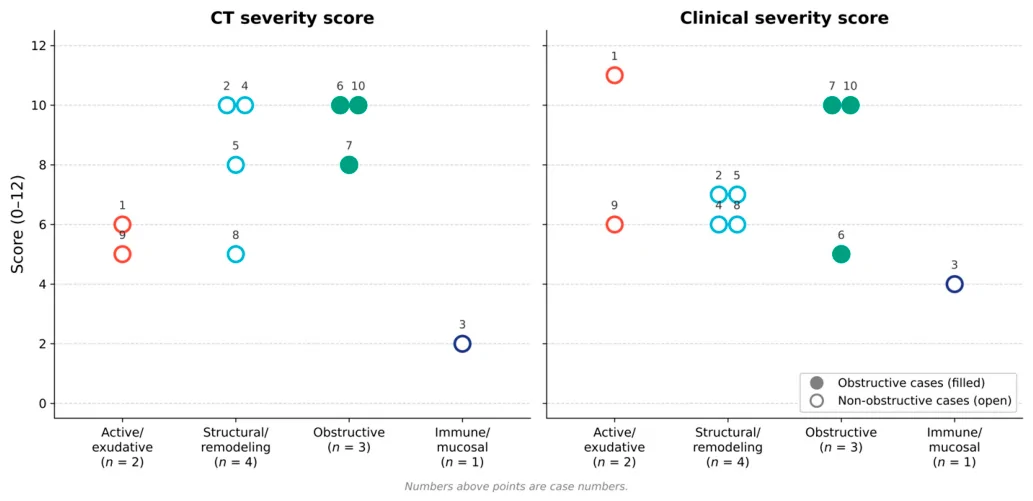
Individual CT severity scores (**left**) and clinical severity scores (**right**) of the 10 cats, grouped by provisional clinicoradiologic pattern. Each symbol represents one cat (numbers indicate case numbers; filled symbols = focal obstruction on CT; open symbols = no focal obstruction). Because groups contained one to four cats, individual observations are shown instead of summary statistics. Colors indicate the provisional pattern, as in Figure 1.

**Figure 3:**
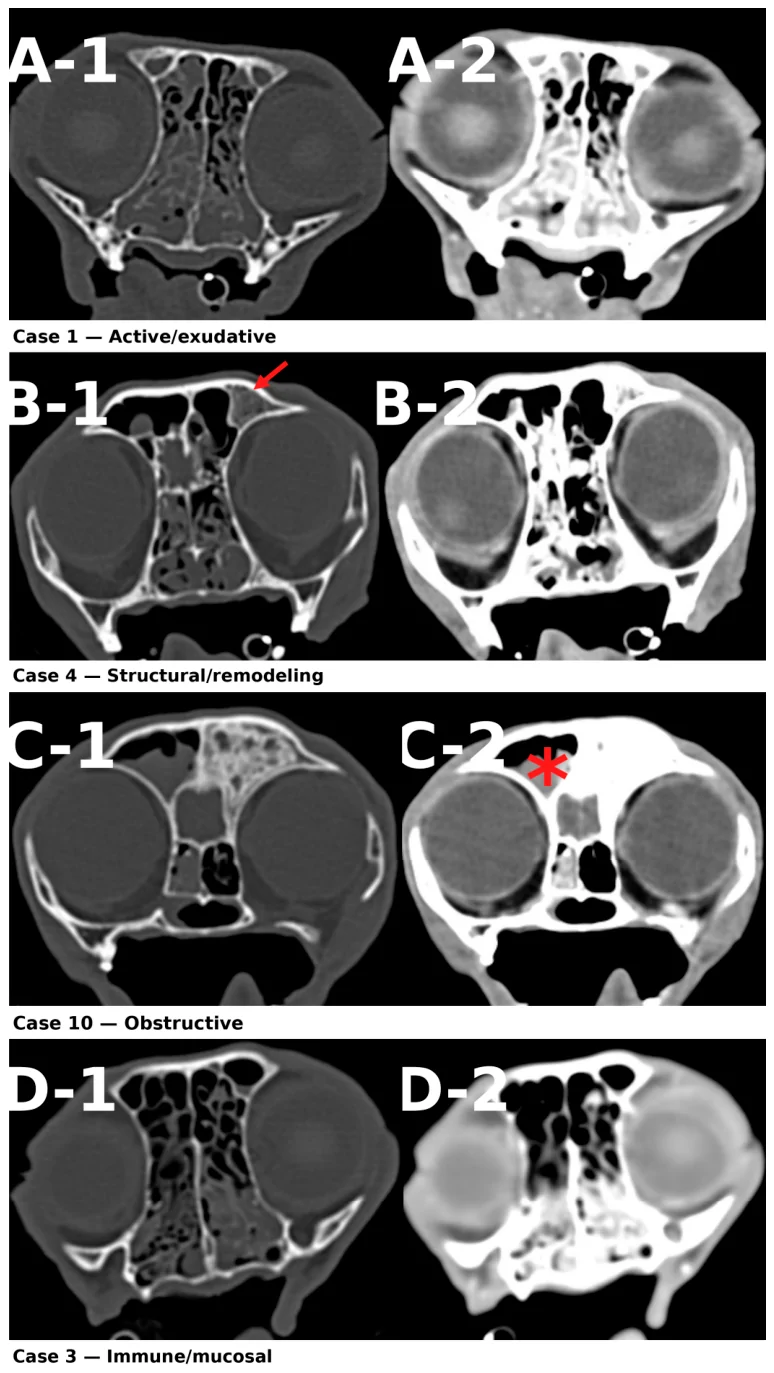
Representative transverse CT images of the nasal cavity and sinuses for the four provisional clinicoradiologic patterns. A bone window (**-1**) and contrast-enhanced soft-tissue window (**-2**) are shown side by side for each pattern (case). (**A**) Active/exudative (Case 1; CT 6/12, clinical 11/12): turbinate architecture is preserved with bilateral intraluminal soft-tissue/fluid attenuation material; no bone remodeling. (**B**) Structural/remodeling (Case 4; CT 10/12, clinical 6/12): severe bilateral turbinate destruction with extensive sinus opacification and marked bone remodeling including dorsal turbinate and left frontal bone hyperostosis; no focal obstructive target. (**C**) Obstructive (Case 10; CT 10/12, clinical 10/12): bilateral frontal sinus opacification, with low-attenuation retained material in the right frontal sinus and endosteal hyperostosis in the left frontal sinus; concurrent bilateral ethmoid lysis. (**D**) Immune/mucosal (Case 3; CT 2/12, clinical 4/12): near-normal turbinate architecture with minimal rostral sinus fluid accumulation; nasal airway preserved. Red arrow indicates frontal bone hyperostosis (**B-1**); red asterisk indicates low-attenuation retained material in the right frontal sinus (**C-2**).

Integrated interpretation of CT findings, clinical severity, the pattern of CT—clinical dissociation, and cytologic data, together with microbiologic data where available, grouped the 10 cats into four provisional clinicoradiologic patterns (Table 2, Figure 2 and Figure 3). Each pattern is summarized briefly below; case-level scores are given in Table 1.

Active/exudative (*n* = 2; Cases 1 and 9). CT scores were 6 and 5 and clinical scores 11 and 6. Both cats had preserved turbinate architecture (turbinate subscore 1), bilateral sinus fluid/exudate (sinus subscore 3) without bony remodeling, and neutrophilic lavage cytology (mild in Case 9). Separately, FHV-1 was detected in Case 9 (virological result, not a cytologic finding). Both cats were considered candidates for medical management.

**Figure 4:**
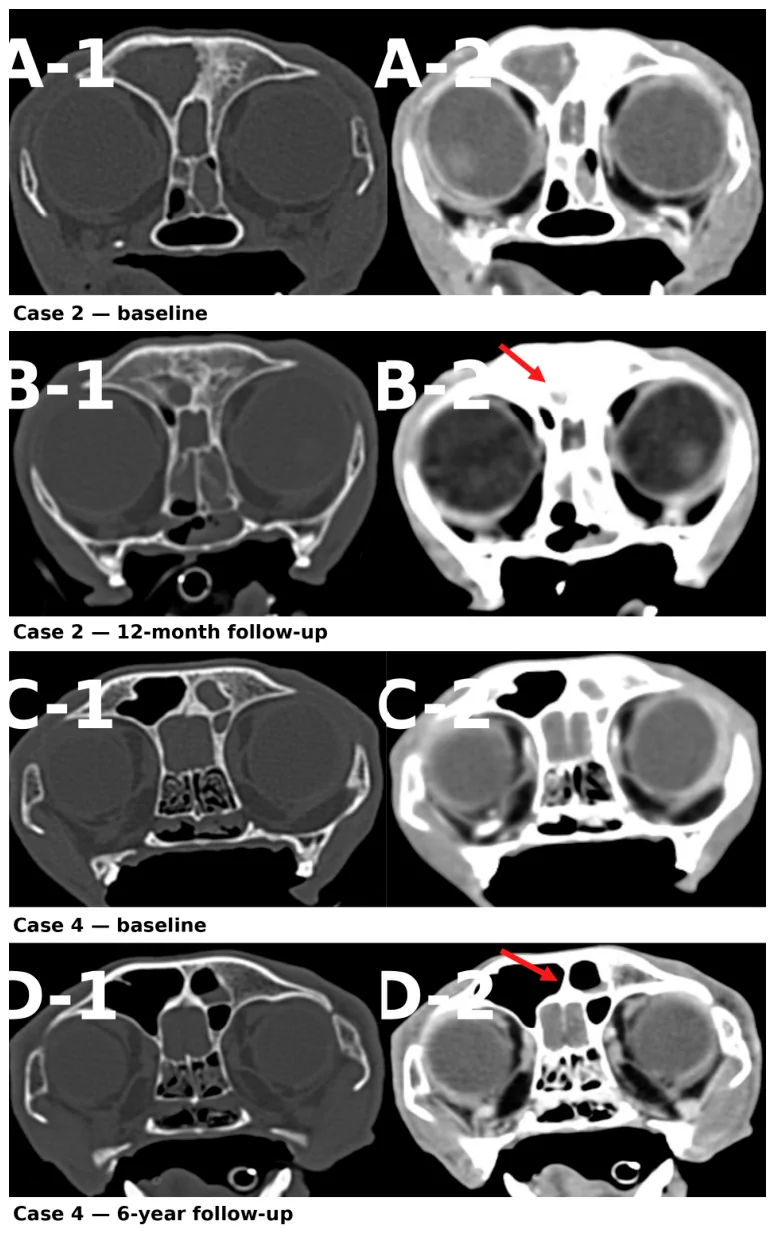
Longitudinal CT changes in the structural/remodeling pattern. (**A**,**B**) Case 2: moderate bilateral turbinate destruction with sinus fluid on the initial CT; progressive turbinate loss with new high-attenuation calcified material in the right frontal sinus on 12-month follow-up CT. (**C**,**D**) Case 4: active bilateral turbinate destruction with sinus opacification on the initial CT; transition to a stable remodeled end-stage with extensive bone proliferation, minimal residual fluid, and preserved sinus aeration on 6-year follow-up CT. A bone window (**-1**) and contrast-enhanced soft-tissue window (**-2**) are shown side by side for each time point. Red arrows indicate new high-attenuation calcified material in the right frontal sinus (**B-2**) and preserved sinus aeration (**D-2**).

Structural/remodeling (*n* = 4; Cases 2, 4, 5, and 8). These cats had turbinate destruction (subscores 2–3) and bony remodeling (subscores 2–3), with CT scores of 5–10 (median 9.0), clinical scores of 6–7 (median 6.5), and CT–clinical differences of +3, +4, +1, and −1. None had focal obstruction, and all were categorized as conservative-management candidates. Serial CT was available for Cases 2 and 4 (Figure 4): Case 2 showed progressive turbinate destruction and new frontal sinus mineralization over 12 months, whereas Case 4 showed a stable remodeled appearance over 6 years, with histopathology showing chronic lymphoplasmacytic rhinitis with osseous proliferation.

**Figure 5:**
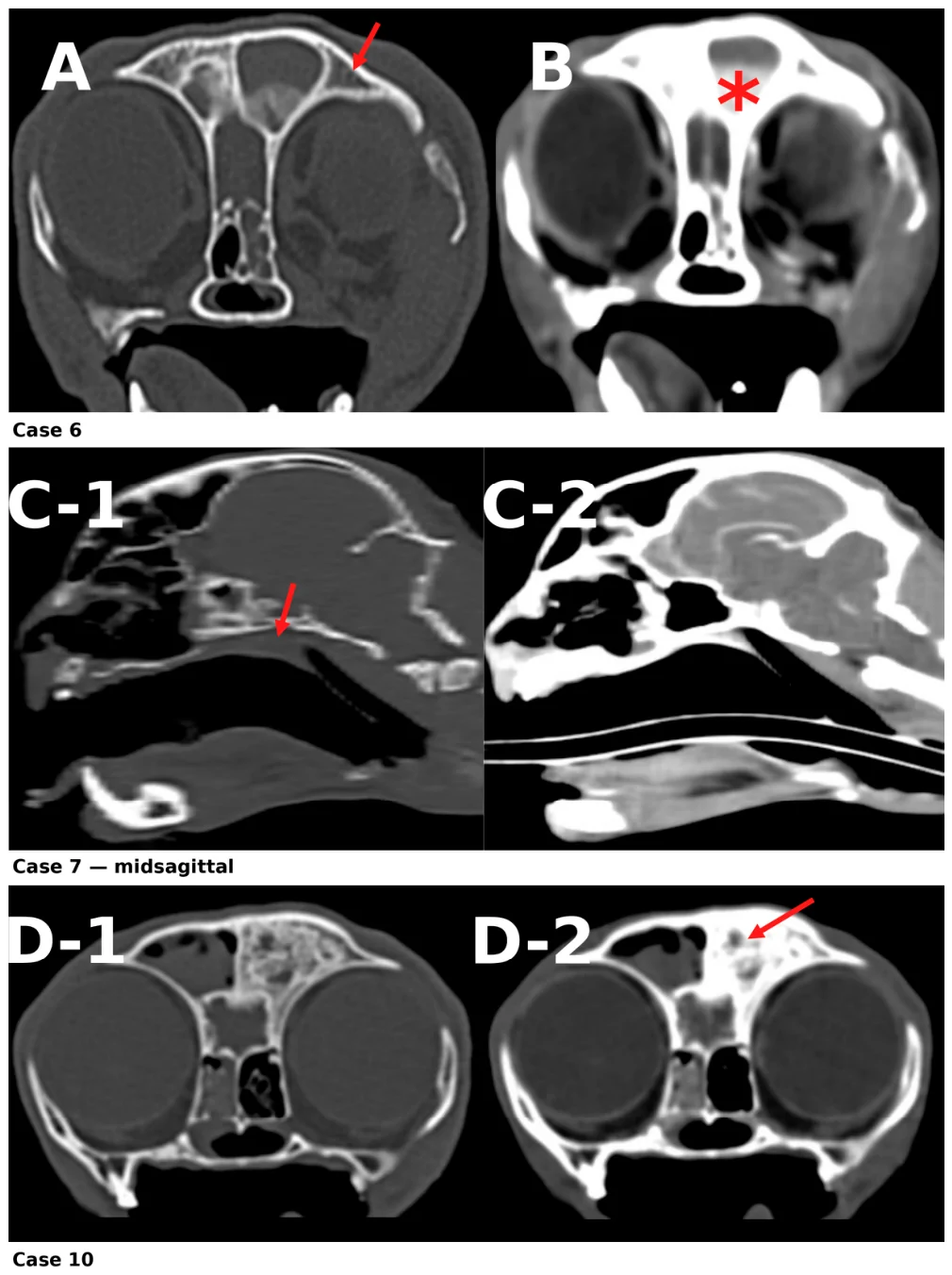
Heterogeneous obstructive mechanisms within the obstructive pattern. (**A**,**B**) Case 6: bone window (**A**) and soft-tissue window (**B**) transverse CT showing high-attenuation (~490 HU) retained material within the left frontal sinus with adjacent endosteal remodeling. (**C**) Case 7: midsagittal reconstruction CT (bone window (**C-1**), soft-tissue window (**C-2**)) showing complete obstruction of the rostral nasopharynx due to dorsal soft palate deviation. (**D**) Case 10: transverse CT (bone window (**D-1**), soft-tissue window (**D-2**)) showing right frontal sinus low-attenuation retained material and left frontal sinus endosteal hyperostosis, with bilateral ethmoid lysis. Red arrows indicate adjacent endosteal remodeling (**A**), obstruction of the rostral nasopharynx by dorsal soft palate deviation (**C-1**), and left frontal sinus endosteal hyperostosis (**D-2**); the red asterisk indicates the high-attenuation (~490 HU) retained material in the left frontal sinus (**B**).

Obstructive (*n* = 3; Cases 6, 7, and 10). CT scores were 10, 8, and 10 and clinical scores 5, 10, and 10, respectively. The obstruction differed among cats (Figure 5): high-attenuation (~490 Hounsfield units) retained material in the left frontal sinus with endosteal remodeling (Case 6); complete obliteration of the rostral nasopharynx by dorsal soft palate deviation (Case 7); and bilateral retained sinus material with frontal sinus hyperostosis and ethmoidal bone lysis (Case 10). Case 6 was categorized as “consider surgical intervention” and Cases 7 and 10 as surgical candidates (conceptual categories; no surgery was performed).

Immune/mucosal (*n* = 1; Case 3). This cat had the lowest CT score (2/12) and a low clinical score (4/12), with preserved turbinates, minimal sinus involvement, and a lymphoplasmacytic cytologic pattern with rare neutrophils and no eosinophils. Being a single cat, it is described only to document an additional exploratory pattern.

### 3.5. Obstruction Status, Candidacy Categories, and CT Severity

By design, focal obstruction on CT defined the obstructive pattern and the “consider surgical intervention” and “surgical candidate” categories; all three cats with obstruction were therefore assigned to these categories, and none of the seven cats without obstruction were (Table 1). This overlap is definitional and is not presented as a finding. The question of interest is the relation to the summed CT severity score, which does not itself define obstruction. Cats with obstruction had numerically higher CT scores (median 10.0; range 8–10) than cats without obstruction (median 6.0; range 2–10), but the ranges overlapped, and no formal comparison was made because of the group sizes (3 vs. 7).

Two cats without obstruction (Cases 2 and 4, both structural/remodeling; clinical scores 7/12 and 6/12) had the maximum CT score of 10/12, whereas Case 7 had a lower CT score (8/12) with nasopharyngeal obstruction. In this small series, therefore, a high summed CT score did not coincide with focal obstruction.

## 4. Discussion

This exploratory pilot study of 10 cats with CRS examined the relationship between CT-based structural severity and clinical severity, described four provisional clinicoradiologic patterns, and explored whether CT evidence of focal obstruction adds information relevant to surgical consideration beyond overall CT severity. The principal observations were: (1) CT and clinical severity scores were widely dispersed, and the correlation estimate was too imprecise to support directional conclusions; (2) integrated review identified four provisional patterns, each based on very few cats and none validated; and (3) the highest CT scores did not coincide with focal obstruction, suggesting that qualitative CT features of drainage obstruction may carry information that a summed severity score does not. These findings are descriptive and hypothesis-generating.

### 4.1. CT–Clinical Dissociation

The weak positive correlation (*r*_s_ = 0.250) with an extremely wide bootstrap 95% CI (−0.558 to 0.885) does not allow any inference about the CT–clinical relationship at *n* = 10 and should not be read as evidence of no relationship. Nevertheless, the dispersion of cases, with CT–clinical differences from −5 to +5, provides descriptive evidence that CT severity and clinical severity can diverge in individual cats. This parallels the poor correlation between imaging severity and symptom severity reported in human CRS [11], where the Lund–Mackay staging system was itself developed in recognition of the unpredictable relationship between CT appearance and symptoms [16]. In cats, Schoenborn et al. found that CT was more sensitive than radiography for defining disease extent in 62 cats with sinonasal disease but not for detecting abnormalities in cats with referable clinical signs [3], suggesting that clinical disease may exist without marked CT changes.

The extreme cases illustrate this divergence: Case 1 had the highest clinical score (11/12) but a CT score of 6/12 with preserved turbinates and exudate-predominant disease, whereas Case 4 had a CT score of 10/12 but a clinical score of 6/12 after 6 years of follow-up. Neither score alone captured disease status in these two cats; this does not establish the underlying mechanisms.

Imaging alone also has limited ability to separate inflammatory subtypes: in a radiographic study of 77 cats, turbinate destruction was associated with neoplasia but was variable and non-specific in chronic rhinitis [21], and combining nasal and medial retropharyngeal lymph node CT features improved discrimination between rhinitis and neoplasia [22]. Whether CT-based severity scores are themselves reliable measurements is addressed next.

### 4.2. CT Technical Considerations and Interpretive Pitfalls

CT-based scoring is also subject to technical artifacts. In experimental canine and feline studies, surrounding fluid reduced turbinate visibility on CT irrespective of bone integrity [23], so poorly visible turbinates in cats with copious secretions do not necessarily indicate osteolysis. This may have affected the turbinate subscores in exudative cases and is a limitation of our scoring. Future studies should use standardized CT protocols, including medium- and high-frequency reconstruction kernels as recommended by Uosyte et al. [23].

### 4.3. Interpretation of the Provisional Patterns

The four patterns are descriptive groupings of one to four cats each that were derived post hoc from the same dataset they describe, using unvalidated scores and non-blinded consensus assessment. They are therefore best regarded as exploratory observations rather than established phenotypes, and the immune/mucosal pattern, based on a single cat, is particularly provisional. They are loosely analogous to the distinction between structural damage and inflammatory activity that endotype-based biomarkers capture in human CRS [7,8,9], but no biomarker data were available here, and cytologic and microbiologic findings contributed to pattern assignment alongside CT and clinical data.

The active/exudative pattern (*n* = 2) combined preserved turbinates and exudate-predominant CT findings with clinical severity exceeding CT severity and neutrophilic cytology; the neutrophilic profile resembles non-type 2 CRS endotypes described in humans [7,9], although this comparison is speculative. The structural/remodeling pattern (*n* = 4) combined turbinate destruction and bone remodeling with CT scores equal to or higher than clinical scores and no focal obstruction, consistent with cumulative structural change outpacing current inflammatory activity. The obstructive pattern (*n* = 3) was defined by focal obstruction, and its mechanisms were heterogeneous (retained sinus material, nasopharyngeal obstruction by soft palate deviation, and bilateral retained sinus material with hyperostosis); it is therefore better regarded as a functional designation than as a single anatomical entity. The immune/mucosal pattern (*n* = 1) had minimal CT changes and lymphoplasmacytic cytology, resembling lymphoplasmacytic rhinitis described in cats [24], but whether it is a distinct entity or a mild form of the non-structural spectrum cannot be determined from one cat.

### 4.4. Longitudinal Observations

Serial CT was available for only two cats with the structural/remodeling pattern, and any inference about temporal evolution is speculative. Case 2 showed progressive turbinate destruction and new frontal sinus mineralization over 12 months, whereas Case 4 showed a stable remodeled appearance over 6 years with declining cytologic inflammation. These contrasting courses suggest that a single CT represents a snapshot along a variable trajectory, in line with the broader airway-remodeling literature in which structural change may persist beyond the inflammatory stimulus [25,26], but our data do not permit conclusions about pattern conversion. Whether progressive mineralization such as that in Case 2 can lead to retained material and obstruction is unknown and would require prospective serial imaging.

### 4.5. Focal Obstruction, CT Severity, and Surgical Consideration

Surgical candidacy categories were defined a priori by CT evidence of focal obstruction or impaired drainage rather than by the summed CT score, and focal obstruction also defined the obstructive pattern. The association between obstruction and candidacy is therefore built into the classification, was not independently demonstrated, and cannot be validated here because no cat underwent surgery. Two different questions should be separated: the summed CT score measures overall structural burden, whereas focal obstruction is a qualitative feature of drainage. Because obstruction was defined independently of the summed score, the two can be compared, and in this series the summed score did not identify the cats with obstruction: two cats with the maximum score of 10/12 had no focal obstruction (Cases 2 and 4), whereas Case 7, with a score of 8/12, had nasopharyngeal obstruction. Classifying cats by CT findings is not inconsistent with this observation: it is the summed score, not CT as a modality, that did not track obstruction or clinical severity, whereas the patterns drew on specific CT features (turbinate, sinus, material, bone, and obstruction) interpreted together with clinical and cytologic findings. Our observation is therefore limited to the descriptive finding that a higher total CT score did not coincide with obstruction in this small series; it does not show that CT is a poor predictor of surgical outcome.

This reasoning mirrors the human CRS surgical guideline, which advises against planning the extent of sinus surgery solely on arbitrary CT thresholds and treats drainage obstruction refractory to medical therapy as a principal surgical indication [17], and the logic of functional endoscopic sinus surgery, whose target is ostiomeatal obstruction rather than inflamed mucosa [27]; meta-analytic data indicate that preoperative CT severity does not reliably predict symptom improvement after surgery [11]. In cats, a recent case report described sustained resolution after frontal sinus trephination and repeated irrigation in a cat with obstructive, treatment-refractory CRS [28]; that cat was not part of the present series. Extrapolation from human guidelines and a single case report to feline practice is speculative.

Case 6 (CT 10/12, clinical 5/12) illustrates that obstructive features may differ in surgical weight: its high-attenuation frontal sinus material was categorized as “consider surgical intervention” because it was not clearly causing drainage impairment, and the cat improved with medical management. In the absence of validated criteria (e.g., attenuation thresholds), such distinctions rely on clinical judgment.

### 4.6. Limitations

This study has important limitations. The sample is very small (*n* = 10) and the patterns contain one to four cats each, so no definitive conclusions can be drawn and the correlation estimate is imprecise. The CT and clinical severity scores were developed for this study, have not been formally validated, and were not assessed for inter- or intraobserver agreement; CT was scored by one radiologist and clinical scores by one clinician, neither of whom was blinded to other findings, and the principal quantitative analysis relates these two new scores to each other. The pattern grouping is a post hoc construct derived from the same data it describes, and the surgical candidacy categories are conceptual, partly circular by design (Section 4.5), extrapolated from human guidelines [17], and unvalidated against surgical outcomes because no cat underwent surgery.

Additional limitations include the retrospective single-center design, heterogeneous diagnostic data (nasal lavage cytology was available for all 10 cats, whereas microbiologic testing and histopathology were available only in a subset), two cats with unrecorded sex, non-standardized CT acquisition, and possible fluid-related underestimation of turbinate visibility in exudative cases [23]. Between-group statistical comparisons were not performed because of the group sizes.

### 4.7. Future Directions

Future studies should (1) validate the CT and clinical scores and test their inter- and intraobserver agreement in a larger, ideally multicenter and prospective cohort (a 95% CI width of about ±0.3 for a Spearman correlation of approximately 0.4 requires roughly 40–50 cases [29]); (2) use blinded assessment and standardized CT protocols, including dual-kernel reconstruction [23]; (3) record outcomes by pattern, including surgical outcomes, to test whether focal obstruction identifies cats that benefit from surgery; and (4) add cytologic, endoscopic, and biomarker data to refine the patterns.

## 5. Conclusions

In this pilot study of 10 cats with chronic rhinosinusitis, CT severity and clinical severity scores diverged in individual cats without a consistent monotonic association, although the correlation estimate was too imprecise for any inference (*r*_s_ = 0.250, 95% CI −0.558 to 0.885). Cats with the highest summed CT scores did not necessarily show CT evidence of focal obstruction, which suggests that obstruction may carry information that a summed score does not; because obstruction also defined the obstructive pattern and the conceptual surgical candidacy categories, this overlap is by design, and no surgical outcomes were available.

The four clinicoradiologic patterns, each based on one to four cats, are provisional and hypothesis-generating, and these findings do not establish a surgical decision-making framework. They support evaluating CT evidence of focal obstruction, rather than overall CT severity alone, as a candidate variable in larger prospective studies with validated scores and surgical outcomes.

## Figures and Tables

**Table 1:** CT scores, clinical scores, CT subscores, provisional pattern, and conceptual surgical candidacy category in 10 cats with chronic rhinosinusitis.

Case	CT (0–12)	Clinical (0–12)	CT–Clin	Turbinate	Sinus	Material	Bone	Obstruction	Provisional Pattern	Surgical Candidacy (Conceptual)
1	6	11	−5	1	3	2	0	No	Active/exudative	Medical
2	10	7	+3	2	3	3	2	No	Structural/remodeling	Conservative
3	2	4	−2	0	1	1	0	No	Immune/mucosal	Medical
4	10	6	+4	3	3	1	3	No	Structural/remodeling	Conservative
5	8	7	+1	2	2	2	2	No	Structural/remodeling	Conservative
6	10	5	+5	2	3	3	2	Yes	Obstructive	Consider
7	8	10	−2	3	2	2	1	Yes	Obstructive	Surgical
8	5	6	−1	2	0	1	2	No	Structural/remodeling	Conservative
9	5	6	−1	1	3	1	0	No	Active/exudative	Medical
10	10	10	0	2	3	2	3	Yes	Obstructive	Surgical

**Table 2:** Characteristics of provisional clinicoradiologic patterns in 10 cats with chronic rhinosinusitis. Surgical candidacy categories are conceptual; no cat underwent surgery.

Provisional Pattern	*n*	CT Median (Range)	Clinical Median (Range)	CT–Clin Median (Range)	Key Distinguishing Features	Surgical Candidacy
Active/exudative	2	5.5 (5–6)	8.5 (6–11)	−3.0 (−5 to −1)	Preserved turbinates; intraluminal exudate; neutrophilic cytology	Medical
Structural/remodeling	4	9.0 (5–10)	6.5 (6–7)	+2.0 (−1 to +4)	Turbinate destruction; bony remodeling; no focal obstruction	Conservative
Obstructive	3	10.0 (8–10)	10.0 (5–10)	0.0 (−2 to +5)	Focal obstruction or impaired drainage; retained/inspissated material	Consider/Surgical
Immune/mucosal	1	2.0	4.0	−2.0	Minimal CT findings; lymphoplasmacytic cytology	Medical

## Data Availability

The data presented in this study are available on request from the corresponding author. The data are not publicly available due to client confidentiality and institutional restrictions on client-owned patient medical records.
